# What is the evidence for abdominal and pelvic floor muscle training to treat diastasis recti abdominis postpartum? A systematic review with meta-analysis

**DOI:** 10.1016/j.bjpt.2021.06.006

**Published:** 2021-07-21

**Authors:** Sandra Gluppe, Marie Ellström Engh, Kari Bø

**Affiliations:** a Department of Sports Medicine, Norwegian School of Sports Sciences, Oslo, Norway; b Department of Obstetrics and Gynaecology, Akershus University Hospital, Lørenskog, Norway; c Faculty of Medicine, University of Oslo, Oslo, Norway

**Keywords:** Diastasis recti abdominis, Exercise, Pelvic floor muscle, Postpartum, Treatment

## Abstract

**Background:**

Diastasis recti abdominis (DRA) affects a significant number of women in the postpartum period.

**Objective:**

To systematically review whether abdominal and pelvic floor muscle (PFM) exercise programs are effective in the treatment of DRA postpartum.

**Methods:**

Electronic search was conducted from inception to March 2020. Randomized controlled trials (RCT) or pilot RCTs that compared abdominal training, PFM training, or a combination of both in at least one arm of the trial were included. The primary outcome was presence of DRA (numbers/percentage) or inter-recti distance (IRD) change. GRADE was used to rate the overall quality of evidence. Pooled effect sizes were expressed as mean difference (MD) with 95% confidence intervals (CI).

**Results:**

Seven RCTs totaling 381 women were included. Two studies comparing transversus abdominis (TrA) training with minimal intervention provided data to be included in a meta-analysis. The results provided very low level quality evidence that TrA training reduced IRD (MD = -0.63 cm, 95% confidence interval: -1.25, -0.01, I^2^ = 0%). Two studies included curl-up exercises as part of their intervention. Level of evidence based on single trials of high risk of bias show very low evidence that curl-up training is more effective than minimal intervention for treating DRA. Similarly, analyses based on single trials provided low to very low quality evidence that PFM training is not more effective than minimal intervention for treating DRA.

**Conclusion:**

There is currently very low-quality scientific evidence to recommend specific exercise programs in the treatment of DRA postpartum.

## Highlights

• Trials investigating abdominal and PFM exercises on DRA have mostly been performed on women with mild DRA.
• The abdominal and PFM exercise programs identified were heterogenous in terms of type of exercises and training dose.
• We found low quality evidence that TrA and curl-up training are more effective than minimal intervention for treating DRA.
• There is low to very low evidence that PFM training is not more effective than minimal intervention.

## Introduction

Diastasis recti abdominis (DRA) is defined as a separation of the two bellies of the rectus abdominis along the midline of linea alba.^1^ The prevalence has been reported to be 60% and 32.5%, six weeks and 12 months postpartum, respectively.^2^ Although this prevalence is high, the exact etiology and pathogenesis of the condition is currently unknown^3^ and there is no consensus whether, for example age, delivery mode, and parity are risk factors for DRA.^4^, ^5^, ^6^, ^7^ In addition to be an aesthetic concern for many women, other suggested consequences are impaired abdominal strength, abdominal, low back, and pelvic girdle pain, and pelvic floor disorders (PFD).^8^, ^9^, ^10^ A recent systematic review^10^ found only weak evidence that DRA severity may be associated with impaired abdominal muscle strength and low back pain severity. In addition to the sparse scientific evidence for consequences of the condition, most studies have included women with mild and moderate DRA only, and there is little knowledge on women with severe diastasis (>5 cm).^8^, ^9^, ^10^

To diagnose and evaluate the presence of DRA the inter-recti distance (IRD) is measured.^11^ Ultrasound, caliper, and palpation are used to measure IRD^12^ with ultrasound having the best reliability with intra- and inter-rater intraclass correlation coefficients >0.9.^13^ There is no consensus on the cut-off point to diagnose DRA.^12^ Candido et al.^6^ classified DRA as mild if IRD was greater than 2.5 cm during a curl-up, and Mota et al.^14^ reported that normal values for IRD in women 6 months postpartum were between 17 mm and 28 mm, with greater values in parous women than in nulliparous women.

The most used exercises recommended by women`s health physical therapists were exercises targeting the transversus abdominis (TrA) (89%) and pelvic floor muscles (PFM) (87%).^15^ However, there is no consensus among health professionals on how to best approach DRA in the primary healthcare system.^16^ In-drawing with contraction of the TrA and internal obliques has been recommended as a gentle exercise to reduce DRA in the postpartum period,^3^^,^^17^ while curl-up has been discouraged. Contradicting common clinical practice, recent results from several experimental studies have found that curl-up leads to an immediate decrease in IRD while in-drawing leads to an increase in IRD.^18^, ^19^, ^20^, ^21^ However, the effect of conducting these exercises over time to reduce IRD is still unknown.

In 2014, Benjamin et al.^3^ presented a systematic review of the effect of abdominal training for DRA. They found only one randomized controlled trial (RCT). They concluded that the effectiveness of abdominal training to prevent or treat women with DRA was undetermined. However, since 2014 there has been an increased scientific interest in DRA and several new RCTs have been published.

The research questions of this systematic review were:

1 Can abdominal training, PFM training, or a combination reduce IRD or prevalence of DRA postpartum?
2 Can abdominal training, PFM training, or a combination improve body image, low back pain, PFD, abdominal muscle strength, and physical function in women with DRA postpartum?

## Methods

This systematic review followed the Preferred Reporting Items for Systematic Reviews and Meta-analyses (PRISMA) Statement.

### Identification and selection of studies

A search was undertaken to identify relevant studies in the electronic databases MEDLINE/Pubmed, Embase, CINAHL, Web of Science, PEDro, and Sport Discus. There was no time limit for publication date. Also, a manual search of reference lists and related studies was conducted. The following search was performed in PubMed on March 18, 2020; (“randomized controlled trial” OR “randomised controlled trial”) AND (“recti abdominis” OR “abdominal rectus diastasis” OR “diastasis recti”) AND (postpartum OR postnatal). Box 1 presents the inclusion criteria for eligible studies. Two independent reviewers screened the titles and abstracts and then evaluated articles available in full text for eligible studies. Any disagreement was solved through discussion until a consensus was reached. Other modalities, e.g. therapeutic taping technique or abdominal binding, could be included in one or more interventions or as a separate intervention.

Box 1 **Inclusion criteria.**

Design

• Randomized controlled trials or pilot randomized controlled trial
• English, Scandinavian, or German language

Participants

• Women with diastasis recti abdominis postpartum
• Primi or multiparous
• Vaginal or caesarean section birth

Intervention

• Abdominal training, pelvic floor muscle training, or a combination of both in at least one arm of the trial

Primary outcome measures

• Presence of diastasis recti abdominis or change in inter-recti distance (cm)

Secondary outcome measures

• Body image, low back pain, pelvic floor disorders, abdominal muscle strength, or physical function

Comparisons

• Other interventions (e.g., abdominal binding), usual care (e.g., general exercise program), or no intervention

Alt-text: Unlabelled box

### Data extraction and quality assessment

We extracted data on participants' characteristics (age, parity, delivery mode), intervention with training dosage (mode of exercise, duration of the exercise period, frequency, training volume, and adherence), DRA cut-off value, measurement method, and primary and secondary outcome measures. In studies with insufficient information, authors were contacted for further details.

The PEDro scale was used to evaluate the risk of bias. The PEDro score ranges from 0 to 10 with higher scores indicating superior methodological quality. A total PEDro score equal to or less than three points are considered poor, score from four to five are considered fair, six to eight are considered good, and nine to 10 are considered excellent.^22^ The PEDro scale has been found to be a valid tool to evaluate methodological quality in clinical trials.^23^^,^^24^ Study selection and data extraction were evaluated independently by two reviewers. For risk of bias, when available we used the PEDro score available in the PEDro website, if not available two reviewers independently rated the trial.

To assess levels of evidence for the meta-analysis and the studies comparing abdominal training to a minimal intervention group, we used the Cochrane Collaboration Grading of Recommendations Assessment, Development and Evaluation (GRADE) approach.^26^ Two authors independently reviewed each study. The GRADEpro GDT^27^ was used to develop a summary of findings table. The quality of evidence for the meta-analysis was downgraded according to the presence of the following: risk of bias (downgraded by one level if more than 25% of the participants were from studies with poor or fair methodological quality), inconsistency of results (downgraded by one level if significant heterogeneity was present by visual inspection or if the I^2^ value was greater than 50%), and imprecision (downgraded by one level if fewer than 70 participants were included in the comparison or downgraded by two levels if participants from pilot studies were included in the meta-analysis). Single randomized trials were considered inconsistent and imprecise (that is, sparse data) and provided “low quality” evidence. This could be further downgraded to “very low” quality evidence if there was also high risk of bias.

### Data synthesis and analysis

Meta-analysis was considered appropriate only for those studies using similar outcome measures, measurement methods, and control groups. The Review Manager 5.4 software, from the Cochrane Collaboration, was used to conduct the meta-analysis. Mean, standard deviation, and sample size from each group were extracted and used to estimate effect sizes. Pooled effect sizes were calculated using fixed effect models and expressed as mean difference (MD) with 95% confidence intervals (CI) in the forest plot. The I squared value if lower than 50% was used to confirm homogeneity among included studies.

When trials were not sufficiently homogeneous, pooling of data via meta-analysis was not performed. Trials were grouped according to the type of intervention (i.e. TrA training, PFM training, and curl-up training). Outcome measures of the individual studies were extracted and difference between groups were expressed as MD and 95% CI.

## Results

### Search results

The systematic literature search identified 31 potential records. In addition, two additional records were identified through personal knowledge. After removing duplicates (*n* = 15) and irrelevant studies (*n* = 6), 12 full-text articles were assessed for eligibility. A total of seven studies were included in this review. No relevant studies were identified through manual search of reference list. Supplemental Online Material shows the flow of studies in the review.

### Studies characteristics

Studies were published between 2016 and 2020 and were conducted in six different countries. Detailed characteristics of included studies are presented in Table 1. Regarding the study design, two studies^28^^,^^29^ were pilot RCTs and five studies^25^^,^^30^, ^31^, ^32^, ^33^ were RCTs. The sample size varied from nine^30^ to 175,^25^ and all women were between 18 and 45 years old. Time since birth for inclusion varied between a couple of days^31^ to three years.^30^ Parity and delivery mode were not reported in two of the included studies^31^^,^^33^ and the others contained a mix between primi and/or multiparous women and women with cesarean section and/or vaginal delivery.^25^^,^^28^^,^^29^^,^^30^^,^^32^ One study^25^ was a secondary analysis of a 2-arms RCT in which the primary aim was to evaluate the effect of PFM training on urinary incontinence.

**Table 1:** Study characteristics.

Authors	Study	Participants (N, age, time PP)	Parity and delivery mode	Cut off value DRA	Main outcome measure	Secondary outcome measures
Walton et al. 2016^30^ *USA*	RCT	*N* = 9 18–45 years 3 months to 3 years PP	Parity not reported. Cesarean section and vaginal delivery (*n* = 1)	Not reported	• IRD measured with ultrasound and caliper 4.5 cm above, at, and 4.5 cm below umbilicus	• ODI • PFDI
Kamel & Jousif 2017^32^ *Egypt*	RCT	*N* = 60 25–35 years 2 months PP	Primi- and multiparous. Vaginal delivery	>2.5 cm measured any place along linea alba during a curl-up	• IRD measured with ultrasound at X-U/2 and U-P/2	• Abdominal muscle strength
Bobowik & Dąbek 2018^31^ *Poland*	RCT	*N* = 40 32.3 ± 5.9 years 0–3 days PP	Parity and delivery mode not reported	≥2 cm	• DRA measured with **palpation** (one finger width = 1.3 cm)	
Tuttle et al. 2018^28^ *USA*	Pilot RCT	*N* = 30 32.03 ± 4.3 years 6–12 weeks PP	Primi- and multiparous. Delivery mode not reported	≥2 finger widths during head lift	• IRD measured with ultrasound 4.5 cm above and below umbilicus during rest and head lift	• PFDI-20 • RDQ
Gluppe et al. 2018^25^ *Norway*	RCT	*N* = 175 29.8 ± 4.1 years 6 weeks PP	Primiparous. Vaginal delivery	≥2 finger widths or a visible protrusion during a curl-up	• DRA measured with palpation 4.5 cm above, at, and 4.5 cm below umbilicus during a modified sit-up Measurements 6 and 12 months PP	
Thabet & Mansour 2019^33^ *Saudi Arabia*	RCT	*N* = 40 22–35 years 3–6 months PP	Parity and delivery mode not reported	>2 cm from umbilicus to 4.5 cm above umbilicus or a visible protrusion	• IRD measured with caliper 4.5 cm above umbilicus during a modified sit-up	• PF10
Keshwani et al. 2019^29^ *Canada*	Pilot RCT	*N* = 32 31 ± 3 years 22 days PP	Primiparous. Vaginal delivery	>2 finger widths at, 2 cm above, 5 cm above, or 3 cm below umbilicus	• IRD measured with ultrasound at umbilicus, 3 cm above, 5 cm above, or 3 cm below umbilicus	• Abdominal muscle strength • PFDI • Body image • IFSAC

DRA, diastasis recti abdominis; IFSAC, inventory of functional status after childbirth; IRD, inter-recti distance; ODI, Oswestry Disability Index; PF10, the Physical Functioning scale; PFDI, Pelvic Floor Distress Index; PP, postpartum; RCT, randomized controlled trial; RMQ, the Roland-Morris Disability Questionnaire; X-U/2, halfway between umbilicus and xiphoid process; U-P/2, halfway between umbilicus and symphysis.

Presence of DRA or IRD change was the primary outcome measure in all included studies. However, the studies used different measurement methods; ultrasound,^28^^,^^29^^,^^32^ palpation,^25^^,^^31^ and both ultrasound and caliper.^30^ The studies measured IRD at different places along linea alba and in different positions, i.e. rest, head lift, and modified curl-up. In addition, the included studies used different cut-off values for DRA, such as 2.0 cm, 2.5 cm, and 2 finger-widths. Secondary outcome measures varied among included studies. Secondary outcome investigated were symptoms of PFD measured with the Pelvic Floor Distress Index (PFDI),^28^^,^^29^^,^^30^ self-report low back disability measured with the Roland Morris Disability Questionnaire (RMQ)^28^ and the Oswestry Disability Index (ODI),^30^ and abdominal muscle strength measured with an isokinetic dynamometer (Biodex)^32^ and with a static trunk flexion endurance test.^29^ In addition, measures of self-reported physical function in the postpartum period^29^^,^^33^ and body image^29^ were assessed.

Interventions, training dosage, and results for primary and secondary outcomes of included studies are presented in Table 2. Many treatment programs contained a plethora of different exercises, modalities, and combinations. Four studies compared the intervention to a minimal intervention group.^25^, ^26^, ^27^, ^28^, ^29^^,^^31^ The control groups included education,^31^ standard information after delivery,^25^ and instruction to maintain normal activity level.^28^ The interventions were performed as home exercise only in some studies^28^^,^^30^^,^^31^ and with individual supervision at the clinic.^32^ Two studies combined daily home training with either supervised weekly group exercise or individual treatment.^25^^,^^29^ The duration of the exercise period varied between six and 16 weeks and total number of repetitions varied from 40^28^ to 210 per week.^29^ Drop-out varied from no drop-out^33^ to 15.5% at 6 months post-test.^29^ Adherence to the exercise programs varied from 73%^29^ to 95%.^28^ No adverse effects were reported. Four studies^28^^,^^31^, ^32^, ^33^ reported a statistically significant difference between groups in reduction of numbers with DRA or decrease in IRD. Of these studies, two^28^^,^^31^ compared a physical therapy intervention to a minimal intervention group (i.e. education). Three studies^25^^,^^29^^,^^30^ did not find a statistically significant decrease in IRD after their training programs.

**Table 2:** Interventions, dosage, drop-out and adherence, results of primary and secondary outcomes, and adverse effects in included studies.

Study	Interventions, number of participants and exercises	Dosage	Drop-out and adherence	Results for DRA presence or IRD in cm, mean ± SD	Results for secondary outcomes	Adverse effects
Walton et al. 2016^30^	**Experimental group (*n*** **=** **5)** • Plank (10 s. on knees or toes) **«Traditional» training (*n*** **=** **4)** • Modified sit-up Both programs contained; • Posterior pelvic tilt • PFM exercises • Exercises for oblique abdominals • Use of abdominal binding during exercise	Duration: 6 weeks Dosage: 3 × 10 repetitions, 3x/week. (Gradually increase repetitions during the period)	Total drop-out: 1 Adherence: Not reported	Post-test: Experimental: IRD: 0.76 ± 0.2 Traditional: IRD: 0.66 ± 0.17 No significant difference in decrease in IRD between groups, at the level at the umbilicus: 0.10 (95% CI: −0.14, 0.34)	• ODI: No significant difference between groups (*p* = 0.569) • PFDI: No significant difference between groups (UDI score; *p* = 0.117)	Not reported
Kamel & Jousif 2017^32^	**Abdominal exercise + NMES (*n*** **=** **30)***Group A* NMES was applied first, followed by the abdominal exercises**Abdominal exercise with abdominal binding (*n*** **=** **30)***Group B* • Sit-up • Reverse sit-up • Reverse trunk twist • U-seat • Respiratory rehabilitation maneuver during exercises	Duration: 8 weeks Dosage: 20 repetitions, 3x/week (Increase with 4 repetitions/week)	Total drop-out: 3 Abdominal exercise (*n* = 2) Abdominal exercise + NMES (*n* = 1) Adherence: Analysis on patients who finished all sessions (same as described in drop-out)	Post-test: Abdominal exercise + NMES: IRD: 1.43 ± 0.38 Abdominal exercise: IRD: 2.09 ± 0.35 Significant difference in decrease in IRD between groups: −0.65 (95% CI: −0.85, −0.46)	• Abdominal muscle strength: Significant difference in group A compared to group B in peak torque (N/m): 5.22 (95% CI: 1.95, 8.5)	Not reported
Bobowik & Dąbek, 2018^31^	**Physical therapy program (*n*** **=** **20)** Part 1: Prone lying for 20 min. Part 2: Three supine abdominal exercises with respiratory maneuver (headlift, sit-up, and “cycling”) Part 3: Education (in/out of bed, lifting the baby, breastfeeding++) (Elastic tape was used once a week) **Minimal intervention group (*n*** **=** **20)** Contained no exercise or tape, only education	Duration: 6 weeks Dosage: Hold: 10 s, 10 repetitions/exercise, every day	Drop-out and adherence not reported	Post-test: Minimal intervention: DRA: 1.68 ± 0.7 Physical therapy: DRA: 0.4 ± 0.23 Significant difference in IRD between groups: −1.28 (95% CI: −1.60, −0.69)		Not reported
Tuttle et al. 2018^28^	**TRA training (*n*** **=** **10)** Home exercise, in-drawing in four different positions with respiratory maneuver **Tape (*n*** **=** **8)** Participants taped themselves with a x-shape, and used the tape for 4–5 days, then 2–4 days off before a new intervention period with tape **TRA+tape (*n*** **=** **5)** Combination of TRA training and kinesiotape **Minimal intervention group (*n*** **=** **7)** Instructed to maintain normal level of activity	Duration: 12 weeks Dosage: 10 repetitions, 4–5 days/week	Total drop-out: 3 TRA (*n* = 1), TRA + tape: (*n* = 1), tape (*n* = 1) Adherence: Average all groups: 79% TRA training only: 95%	Post test^1^ TRA: IRD: 1.34 ± 0.37 Minimal intervention: IRD: 2.1 ± 0.99 Close to a significant difference in IRD between groups: −0.76 (95% CI: −1.53, 0.01) Significant better decrease in IRD at rest and during head lift in the groups with TRA training compared to control/tape (post hoc t-test)	• PFDI-20: No significant difference between groups (*p* >0.05). • **RMDQ:** No significant difference between groups (*p* >0.05).	Not reported
Gluppe et al. 2018^25^	**Postpartum training program (*n*** **=** **87)** Weekly supervised exercise class with strength training of PFM in 5 different positions in addition to strength exercises for abdominal,^2^ back, arm, and thigh muscles. Daily PFM training at home **Minimal intervention group (*n*** **=** **88)** Received only standard information about exercise postpartum	Duration: 16 weeks Dosage: 3 × 8–12 repetitions. PFM training daily, group training once a week	6 months Total drop-out: 13; intervention (*n* = 10), control (*n* = 3) 12 months Total drop-out: 5; intervention (*n* = 1), control (*n* = 4) Adherence: Postpartum training program: 80% adherence to training for 96% of women	Post-test 6 months: Exercise: DRA, 43.7% Minimal intervention: DRA, 44.3% 12 months: Exercise: DRA, 41.4% Minimal intervention: DRA, 39.8% No significant difference between groups 6 months PP, (RR: 0.99 [0.71, 1.38]) or 12 months PP, (RR: 1.04 [0.73, 1.49])		Not reported
Thabet & Alshehri 2019^33^	**Deep core stability-strengthening program** (+ traditional exercises) **(*n*** **=** **20)** *Group A* Use of abdominal binding, respiratory maneuver, PFM exercises, plank and isometric abdominal contraction **Traditional abdominal exercises (*n*** **=** **20)***Group B* Static abdominal contractions, posterior pelvic tilt, reverse sit-up, trunk twist and reverse trunk	Duration: 8 weeks Dosage: 3 × 20 repetitions, 3/week	No drop-out Adherence: Not reported	Post-test: Deep core training: IRD: 2.01 ± 0.07 Traditional exercises: IRD: 2.37 ± 0.11 Significant difference in IRD between groups = −0.36 (95% CI: −0.42, −0.30)	• PF10: Significant difference in group A compared to group B: 5.25, *p* = 0.0001	Not reported
Keshwani et al. 2019^29^	**Exercise therapy (*n*** **=** **8)** Weekly individual sessions and daily home exercise including exercises for isolated activation of TRA **Abdominal binding (*n*** **=** **8)** Wear binding during waking hours **Combination therapy (*n*** **=** **8)** Combination of exercise therapy and abdominal binding **Minimal intervention group (*n*** **=** **8)** Contained no intervention or education	Duration: 12 weeks Dosage: 3 × 10 repetitions, 7x/week	6 months Total drop-out: 5; exercise therapy (*n* = 2), control (*n* = 1), exercise therapy+abdominal binding (*n* = 2) Adherence: Exercise therapy; 73% (home exercise) and 10/12 of the weekly sessions Abdominal binding; 60% Combination group was similar to the interventions delivered alone	Post-test: 6 months Exercise therapy: IRD: −0.93 ± 0.88 Abdominal binding: IRD: −1.34 ± 0.34 Combination: IRD: −1.24 ± 0.73) Minimal intervention: IRD: −1.31 ± 1.08 No significant difference between groups. When comparing exercise therapy to control, no significant difference between groups was found: −0.38 (95% CI: −1.45, 0.68)	• Abdominal muscle strength: Positive effects (Cohen`s d (d)= 0.5–0.7) in the exercise and combination groups. • PFDI: No effects in any groups • Body image: Positive effects (*d* = 0.2–0.5) in the abdominal binding alone and combination groups. • IFSAC No effects (*d* = 0.0–0.3) in any groups	Not reported

DRA, diastasis recti abdominis; IFSAC, inventory of functional status after childbirth; IRD, inter-recti distance; NMES, neuromuscular electrical stimulation; ODI, Oswestry Disability Index; PFDI, Pelvic Floor Distress Index; PF10, the Physical Functioning scale; PFM, pelvic floor muscle; PP, postpartum; RCT, randomized controlled trial; RMDQ, the Roland-Morris Disability Questionnaire; TrA, transversus abdominis; UDI, Urinary distress inventory (1/3 subscales of PFDI).

1 Results are presented for measurements at the level at the umbilicus at rest.

2 The weekly exercise class included 3 sets of 8–12 contractions of each of the following abdominal exercises; draw-in (on all fours), draw-in (prone), half-plank, side-plank, oblique sit-up or sit-up.

### Risk of bias

Supplemental Online Material shows the scores on the PEDro Rating scale. There were no disagreements between the assessors in the evaluation process. The PEDro score varied between four and eight points.

### Primary outcomes

#### TrA training

Four RCTs included TrA training among other exercises,^28^^,^^29^^,^^30^^,^^33^ and two studies reported a significant reduction in IRD.^28^^,^^33^

**Figure 1:**
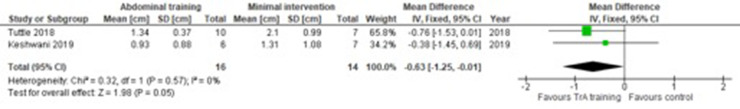
Forest plot on the effect of abdominal training on inter-recti distance in women with diastasis recti abdominis.

Two pilot studies^28^^,^^29^ provided data on the same outcome measure (i.e. IRD) and compared exercises (i.e. TrA) versus a minimal intervention group (i.e. education). Meta-analysis showed TrA training was effective in reducing IRD (2 trials; *n* = 30; MD = −0.63; 95% CI: −1.25, −0.01; I^2^ = 0%) compared to a minimal intervention (Fig. 1). The quality of evidence for the meta-analysis was downgraded to very low due to risk of bias, inconsistency, and imprecision (Table 3).

**Table 3:** Summary of findings table.

N° of studies	Quality assessment			N° of participants		Mean Difference (95% CI)	Quality
	Risk of bias	Inconsistency	Imprecision	Intervention group	Control group		
***IRD (Ultrasound measure): TrA training* versus *minimal intervention group (follow up 12 weeks)***							
2 Pilot RCTs^28^^,^^29^	Serious*	Non-serious	Very serious⁎⁎	16	14	−0.63 (−1.25, −0.01)	⨁○○○ Very low

⁎ Downgraded by one level due to one study classified as fair methodological quality.

⁎⁎ Downgraded by two levels because participants were from pilot studies.

#### PFM training

None of the seven RCTs used PFM training as the sole intervention. Along with several abdominal exercises, PFM training was included in the training programs in three studies.^25^^,^^30^^,^^33^ In these studies IRD was measured with palpation,^25^ caliper,^33^ and caliper and ultrasound.^30^ Sample size varied between 175,^25^ 40,^33^ and nine.^30^ Gluppe et al.^25^ compared the postpartum training program including PFM training with a minimal intervention (i.e., education) and found similar rates in both groups of participants with DRA at 6 and 12 months. Walton et al.^30^ showed that a core strengthening program including PFM training was not superior to plank exercise program in reducing IRD (MD = 0.10 cm, 95% CI: −0.14, 0.34). Thabet and Alshehri^33^ found that a deep core stability training including PFM training was more effective in reducing IRD (MD = −0.36 cm, 95% CI: −0,42, −0.30) compared to a traditional abdominal exercise program. Overall, our findings showed low to very low quality evidence that PFM training is not more effective than minimal intervention for treating DRA. Level of evidence was based on single trials of high risk of bias.

#### Curl-up training

Two studies included curl-up exercises as part of their intervention. IRD was measured with palpation^31^ and ultrasound,^32^ and the sample sizes were 40^32^ and 60.^32^ Bobowik and Dąbek^31^ found that the physical therapy program, which included curl-up training, was more effective in reducing IRD (MD = −1.28 cm, 95% CI: −1.60, −0.69) compared to the minimal intervention group. Kamel and Jousif^32^ showed that abdominal exercises with neuromuscular electrical stimulation was more effective in reducing IRD (MD = −0.65, 95% CI: −0.85, −0.46) compared to abdominal exercises only. Our findings show very low evidence that curl-up training is more effective than minimal intervention for treating DRA. Level of evidence was based on single trials of high risk of bias.

### Secondary outcomes

There were few reports on our selected secondary outcomes in the published RCTs (Table 2). One study reported a positive effect on body image and two studies measuring abdominal muscle strength reported positive effects.**^29^****^,^****^33^** No statistically significant effects were found on low back pain or PFD.**^28^****^,^****^29^****^,^****^30^** Two studies found contradictory results in self-report of physical function postpartum.^29^^,^^33^

## Discussion

This systematic review included seven RCTs, of which two were pilot studies, on the effect of abdominal training or PFM training, or a combination, on DRA or IRD in the postpartum period. Unfortunately, a huge heterogeneity in the use of outcome measures, measurement methods and locations, the definition of cut-off point for diastasis, and content of the interventions did not warrant a meta-analysis for all the included RCTs and for secondary outcome measures. Based on meta-analysis of two RCTs,^28^^,^^29^ this systematic review found **very** low-level evidence that TrA training may decrease IRD. So far, the results from RCTs are contradictory, and there is still not enough evidence to recommend any specific physiotherapeutic exercise programs for DRA.

The methodological quality of the RCTs varied between four and eight on the PEDro scale.^23^ Common methodological flaws identified were lack of concealed allocation, blinding of participants and therapists, and intention to treat analysis. These factors are of great importance for the internal validity of intervention studies.^34^ While blinding of assessors was done in all except one study,^31^ blinding of therapists and participants is almost impossible in exercise studies.^35^ Therefore, bias due to participants’ and therapists’ expectations and attitudes to the treatment cannot be excluded.^35^ Another flaw was the very small sample size in some studies^28^^,^^29^^,^^30^ which may have caused a type Ⅱ error. However, these flaws were equally distributed in studies with positive and negative results and can therefore not be used to explain either findings.

Abdominal training in the studies included in the meta-analysis consisted of TrA exercises. Although these two studies^28^^,^^29^ showed a significant decrease in IRD when comparing abdominal training to a minimal intervention group, the quality of evidence was considered very low. Therefore, the results of the meta-analysis should not be implemented in clinical practice guidelines. In addition, we also question the clinical relevance of the pooled mean difference of −0.6 cm and wide CIs.^28^^,^^29^

A common flaw in RCTs is an inadequate description of the intervention.^36^ Important factors to report for analyses of interventional quality should include type of exercise, frequency, intensity, duration of training, and adherence.^37^^,^^38^ The exercise programs for DRA can be classified as strength training. Recommendation for strength training in the postpartum period is the same as for the adult population^39^ and includes 60–70% of 1-repetition maximum (1-RM) (muscular endurance: ˂50% of 1-RM), 2–4 set (muscular endurance: ≤2), 8–12 repetitions (muscular endurance: 15–20), 2–3 days per week, with a gradual increase in training progression.^40^ The number of sets, repetitions, and days per week in the included studies` exercise interventions varied from 1 to 3,^30^^,^^32^ 8–20,^25^^,^^33^ and 1–7,^25^^,^^31^ respectively. The training dosage and adherence varied in those studies reporting no effect of exercise intervention, but adherence was generally high in all studies.^25^^,^^29^^,^^30^ We consider that the PFM training, but not the direct abdominal training, in Gluppe et al.^25^ fulfilled the recommendations for strength training. In the study by Walton et al.^30^ intensity was not reported, and the duration was only six weeks. Hence, the absence of effect of the abdominal- and/or PFM training programs in the studies in IRD/DRA may be due to low training dosage.

### PFM training

PFM training was part of the exercise program^30^^,^^33^ or the primary intervention.^25^ Out of four RCTs reporting a positive effect on IRD or prevalence of DRA, only Thabet and Alshehri^33^ included PFM training. Out of three RCTs reporting no effect on IRD and prevalence on DRA, two studies included daily PFM training^25^ or PFM training as part of the exercise in both intervention groups.^30^ Therefore, it is reasonable to conclude that PFM training was not the exercise causing the effect found in four studies reporting effect on IRD or prevalence of DRA. Also, if PFM training has a positive effect, this should have been found in the study^25^ where the focus was on this muscle group. The latter is supported by the findings in several studies^20^^,^^21^^,^^41^^,^^42^ showing a significant widening, not narrowing, of the IRD during a single PFM contraction. PFM training is first-line treatment for urinary incontinence in women^43^ and has also shown to be effective in the early postpartum period.^44^ Although the immediate effect of contracting the PFM has shown a widening of the IRD, this widening is minimal (mm)^21^^,^^42^ and probably does not influence DRA. Women with DRA should therefore not be discouraged from doing PFM training in the postpartum period.

### TrA training

Of the four studies reporting a positive effect on IRD or prevalence of DRA, two studies included mainly exercises targeting TrA.^28^^,^^33^ One study^33^ did not include a minimal intervention group, and another^28^ had a very small sample size. In contradiction, two studies^29^^,^^30^ found no effect of TrA training but their results were not compared with a minimal intervention group and included women who may not be classified as having DRA,^30^ or had a very small sample size.^29^ Hence, there is very low quality evidence that TrA training is more effective than minimal intervention for treating DRA. Experimental studies have shown that TrA contractions widen the IRD,^19^^,^^21^^,^^41^ and the effect of training TrA over time may therefore also be questioned.

### Curl-up training

Two of the RCTs reported a positive effect on IRD or prevalence of DRA from curl-up exercises in women 0–3 days postpartum^31^ or two months postpartum.^32^ Kamel andYousif^32^ did not include a minimal intervention group. Due to the natural decrease in IRD postpartum^2^ and also the inclusion of other elements in the training protocol (e.g. neuromuscular electrical stimulation, prone lying) it is not possible to conclude whether curl-ups or twisted curl-ups are effective in the decrease of IRD or prevalence of DRA. Several experimental studies have shown that curl-up leads to an immediate decrease in IRD.^41^^,^^42^ A possible explanation for why curl-up might be more effective than PFM or TrA training is that the insertion and origin of TrA means that a contraction of the muscle pulls away from the midline. Because there is a co-contraction of TrA in a maximal voluntary contraction of the PFM,^45^ this may explain why contraction these muscle groups may increase the IRD. There is a need for more basic research to understand the influence of the abdominal muscles on the linea alba and IRD.

Regarding the secondary outcomes of this review, a lack of effectiveness was found on low back pain and PFD.^28^^,^^29^^,^^30^ No association between DRA and PFD in the postpartum period has been found in studies of other designs.^2^^,^^45^^,^^46^^,^^47^ Our results indicate that some of these exercise programs might improve body image, physical function, and abdominal muscle strength.^29^^,^^32^ However, whether these complaints are caused by or related to DRA is currently unclear.^2^^,^^8^

A limitation of our review is the inclusion of studies published in English, German, or Scandinavian languages only. Four of seven included studies did not involve a minimal intervention group. This is considered a limitation because of the natural remission of DRA until at least 12 months postpartum.^2^ Because intervention protocols often combined different exercises and modalities, it is not possible to conclude which specific exercises may have caused the effect in some of the RCTs.^28^^,^^29^^,^^30^, ^31^, ^32^, ^33^ In addition, with the results of experimental studies in mind,^20^^,^^41^ inclusion of different types of exercises as part of the same intervention may have led to the effect of the exercises cancelling each other. Physical therapists should be cautious in promising effect of, or advocating specific exercises, in the treatment of DRA. There is an urgent need for larger, high-quality RCTs with designs to treat women with DRA, investigating the effect of single exercises on IRD and DRA in the post-partum period. As all the RCTs so far have included women with mild/moderate DRA only,^25^^,^^28^^,^^29^^,^^30^, ^31^, ^32^, ^33^ there is also an urgent need to conduct RCTs in women with severe diastasis.

## Conclusion

Our findings show very low evidence that TrA and curl-up training are more effective than minimal intervention for treating DRA. There is low to very low evidence that PFM training is not more effective than minimal intervention. There is currently very low-quality scientific evidence to recommend specific exercise programs in the treatment of DRA postpartum.

## Conflicts of interest

There was no conflict of interest in this study.

## Appendix Supplementary materials

Supplementary online material Flow chart of systematic literature search for included studies and Assessment of risk of bias with PEDro scale Image, application 1
