# Supplementary material for: What is the evidence for abdominal and pelvic floor muscle training to treat diastasis recti abdominis postpartum? A systematic review with meta-analysis
Source: Braz J Phys Ther. 2021 Jul 21;25(6):664–75. doi: 10.1016/j.bjpt.2021.06.006 (PMC8721086; doi:10.1016/j.bjpt.2021.06.006)
Supplement: Supplementary file 1 — Supplementary online material Flow chart of systematic literature search for included studies and Assessment of risk of bias with PEDro scale [file mmc1.docx]

**Supplementary online material:** Flow chart of systematic literature search for included studies.

Records after duplicates removed
(n = 18)

Records screened
(n = 18)

Records identified through database searching (n = 31)

- PubMed: (n = 4)
- Web of Science (n = 5)
- PEDro (n = 10)
- SPORTdiscus (n = 2)
- Embase (n = 6)
- CINAHL (n = 4)

## Identification

## Eligibility

## Included

## Screening

Additional records identified through personal knowledge
(n = 2)

Irrelevant records excluded
(n = 6)

e.g. surgical techniques

Full-text articles excluded, with reasons
(n = 5)

- Language (n = 2)

-Portuguese

-Persian (Farsi)

- Excluded women with DRA (n= 1)
- Study design (n = 1)
- Included men (n = 1)

Full-text articles assessed for eligibility
(n = 12)

Studies included in qualitative synthesis
(n = 7)

| ***Supplemental online material:*** *Assessment of risk of bias PEDro scale* |
| --- |

|  | **Walton**  **2016** | **Kamel**  **2017** | **Bobowik**  **2018** | **Tuttle**  **2018** | **Gluppe**  **2018** | **Thabet**  **2019** | **Keshwani**  **2019** |
| --- | --- | --- | --- | --- | --- | --- | --- |
| Eligibility criteria^a^ | Yes | Yes | No | Yes | Yes | Yes | Yes |
| Random allocation | 1 | 1 | 1 | 1 | 1 | 1 | 1 |
| Concealed allocation | 0 | 0 | 0 | 1 | 1 | 0 | 0 |
| Baseline comparability | 0 | 1 | 1 | 1 | 1 | 1 | 0 |
| Blinded subjects | 0 | 0 | 0 | 0 | 0 | 0 | 0 |
| Blinded therapists | 0 | 0 | 0 | 0 | 0 | 0 | 0 |
| Blinded assessors | 1 | 1 | 0 | 1 | 1 | 1 | 1 |
| Adequate follow-up | 1 | 1 | 0 | 0 | 1 | 1 | 1 |
| Intention-to-treat analysis | 0 | 0 | 0 | 0 | 1 | 1 | 0 |
| Between group comparisons | 1 | 1 | 1 | 1 | 1 | 1 | 1 |
| Point estimates and variability | 1 | 1 | 1 | 1 | 1 | 0 | 1 |
| **Total** | **5/10** | **6/10** | **4/10** | **6/10** | **8/10** | **6/10** | **5/10** |
| PEDro, Physical therapy Evidence Database; 1 = yes, 0 = no  ^a^Not included in total score | | | |  | | | |
